# CHCl^·–^ activates oxygen-containing gases: Uncovering intrinsic reaction nature from a multiscale theoretical investigation

**DOI:** 10.1016/j.isci.2026.117520

**Published:** 2026-09-16

**Authors:** Junxi Liang, Jie Gao, Xinjie Wang, Yalu Zhang, Xiaoyan Bai, Nanzhen Yuan, Huiqiong Wu, Yan Shen, Pen Jin, Yanbin Wang, Qiong Su

**Affiliations:** 1 Key Laboratory of Environment-Friendly Composite Materials of the State Ethnic Affairs Commission, Gansu Province Engineering Research Center for Biomass Functional Composite Materials, Key Laboratory for the Utilization of Environment-Friendly Composite Materials and Biomass in Universities of Gansu Province, Gansu Province Research Center for Basic Sciences of Surface and Interface Chemistry, College of Chemical Engineering, Northwest Minzu University, Lanzhou, Gansu 730030, China; 2 College of Materials Science and Engineering, Nanjing Tech University, Nanjing, Jiangsu 211800, China

**Keywords:** oxygen containing molecules, chemical selectivity, reaction mechanism, multi-scale computational simulation

## Abstract

Understanding the ionic reactivity of CHCl˙^–^ anions is essential for modeling chemical processes in interstellar media, plasmas, and planetary atmospheres. Using multiscale computational simulations, this work examines the adsorption and reaction behaviors of eight oxygen-containing gases (O_2_, CO, CO_2_, NO, N_2_O, NO_2_, COS, SO_2_) on CHCl˙^–^ clusters. Our findings reveal that electrostatic interactions dominate system stabilization over dispersion forces. The O_2_ environment strongly induces activation of the CHCl˙^–^ anion, while COS exhibits minimal reactivity. Bond activation occurs across O-O, C-O, C-S, N-O, N-N, and S-O linkages, with binding strengths following O_2_ > NO_2_ > SO_2_ > CO. Although CO_2_, NO, and N_2_O interactions are thermodynamically limited at room temperature, they become accessible upon heating. These results demonstrate that anion-molecule reactivity emerges from coupled mass transport, electrostatic compatibility, and bond lability, thereby advancing predictive frameworks for radical-mediated chemistry.

## Highlights

• Electrostatics—not dispersion—governs CHCl^·–^ anion stabilization with O-containing gases
• CHCl^·–^ maximally activates O_2_, COS shows weakest reactivity due to C/S-site preference
• CHCl^·–^ cleaves O–O, C–O, N–O bonds; reactivity order: O_2_ > NO_2_ > SO_2_ > CO
• CO undergoes C–C coupling with CHCl^·–^, CO_2_/NO/N_2_O need elevated temperatures

## Graphical abstract

**Figure:**
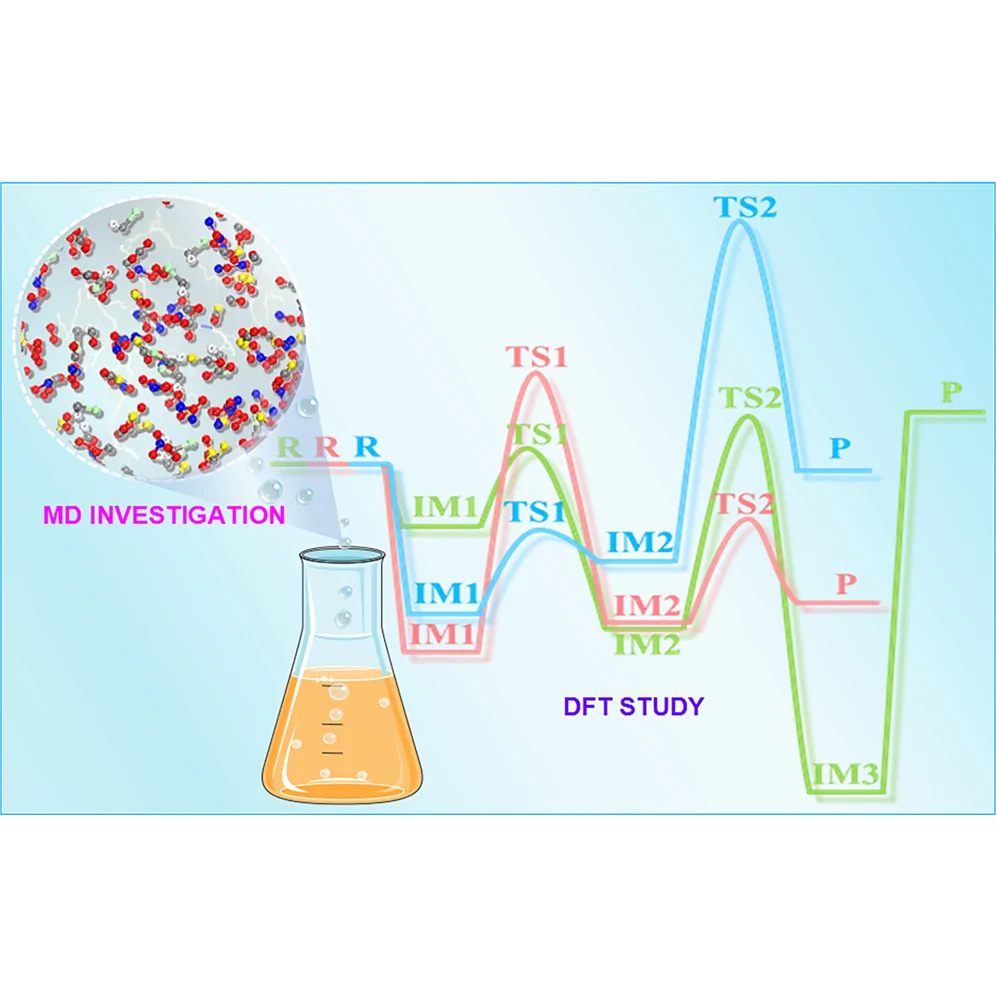

## Teaser

Catalysis; Physical chemistry; Computational chemistry

## Introduction

Carbene radical anions have been extensively studied in gas-phase molecular transformations due to their dual features as both unpaired electrons (radicals) and extra electrons (anions).^1^^,^^2^^,^^3^ For this kind of highly reactive species, studies by Parker and Bethell et al. and later the McDonald group reported their synthesis from the production of diazoalkanes.^4^^,^^5^ Subsequently, Villano et al. elucidated the mechanisms of halogen-substituted variants in the gas phase.^6^ Through this research, novel reaction pathways can be revealed, including electron transfer, insertion, and addition-elimination. Such discoveries significantly expanded free radical chemistry. For example, Betrand et al. reviewed how carbenes stabilize main-group radicals.^7^ Bruin and co-workers explored carbenoid radical-mediated reactions, such as HAT and cyclopropanation.^8^ Among these species, the CHCl˙^–^ has attracted specific attention. The Nibbering group conducted a detailed investigation into its reactions with a variety of methyl halides in 1994–1996, proposing an S_N_2 mechanism.^9^^,^^10^ Building on Nibbering’s work, Villano et al. expanded these reactions in 2009.^11^^,^^12^ Nevertheless, in their experiment, the structures of the ionic and neutral products were often inferred. This inferential approach limits discovery. It constrains new reaction channels and hinders the development of a unified theoretical framework. Later, Li et al.’s theoretical prediction of the products for the CHCl˙^–^ + CS_2_ reaction suggests that Villano et al. overestimated the yields of CClS^−^ and HCS.^13^ Clearly, precise characterization is essential. We need accurate mapping of reaction pathways, transition states, energy barriers, and product distributions. This will better elucidate mechanisms and interpret experimental data. Moreover, such detailed understanding may also help to unravel the synergistic interplay between electron transfer and radical reactivity, offering new perspectives on complex ion-molecule reactions.

**Figure 1:**
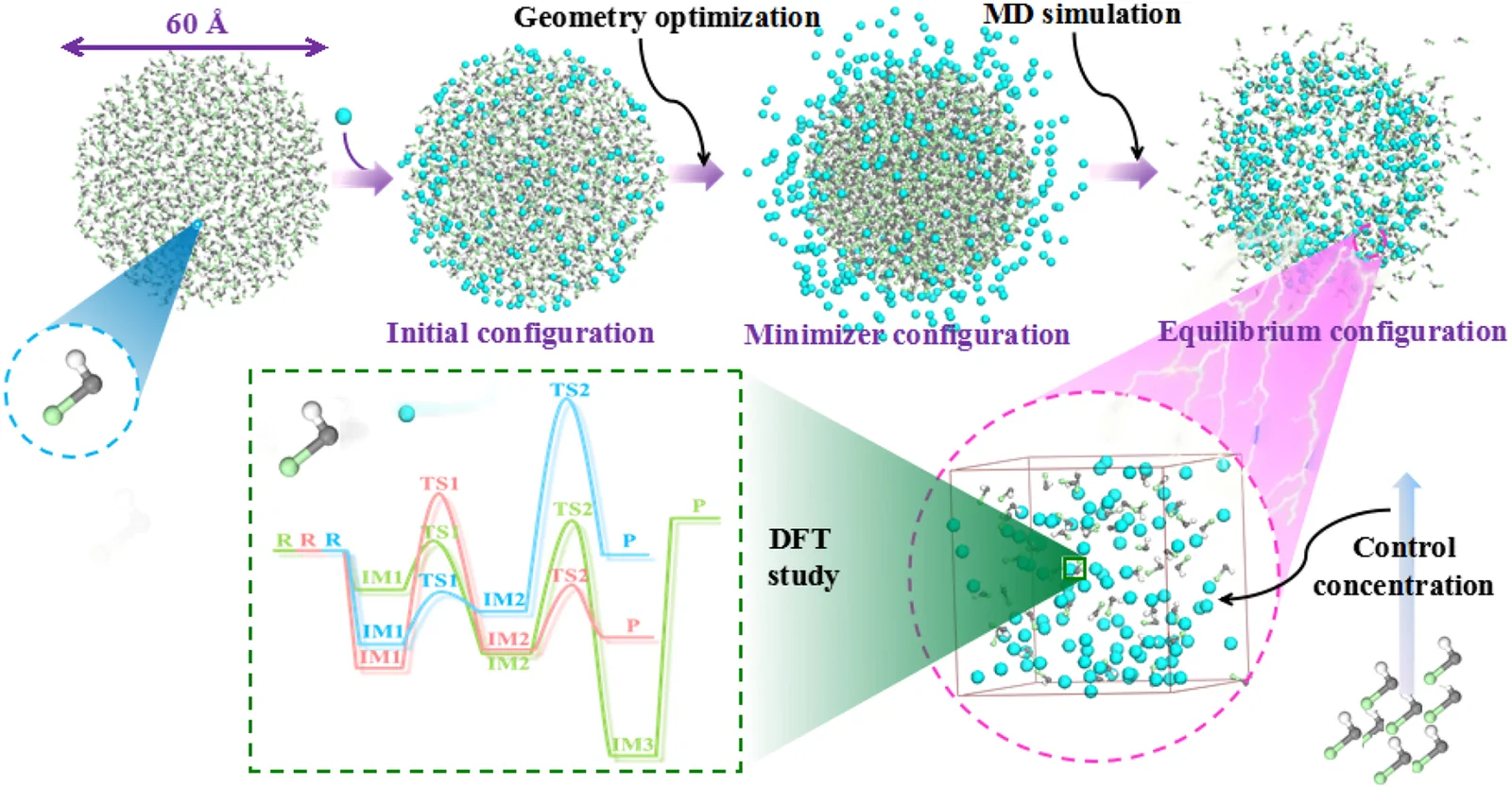
The process of the simulation and calculation

The systematic study of the behavior of CHCl˙^–^ reactions via multi-scale theoretical simulations—spanning cluster models, molecular dynamics (MD), and high-precision density functional theory (DFT) calculations—establishes a robust framework bridging static structural features, dynamic evolution, and atomistic mechanisms.^14^^,^^15^^,^^16^ Crucially, while DFT identifies the stationary points on the potential energy surface, MD captures the temporal evolution of reactive trajectories and thermal fluctuations inherent to the gas-phase cluster microenvironment. This dynamic perspective reveals how conformational heterogeneity modulates reactive site accessibility through transient weak interactions. Such entropic insights are unattainable from static calculations alone. Consequently, by employing this integrated approach, we clarify how the gas-phase microenvironment governs reaction selectivity and product branching ratios. This offers a rational basis for enhancing reactivity and efficiency in complex chemical systems. Noted that many reactive oxygen species originate from O_2_^17^; therefore, the oxygen-containing molecules presented here can lead to the generation of reactive oxygen, nitrogen, and sulfur due to their reactive radical nature. Prior work showed that oxygen-transfer reactions extracting O atoms from CO, CO_2_, NO, N_2_O, and O_2_ favor CO selectivity.^18^ Very recently, Chan et al. prepared cMOFs that exhibit higher NO selectivity over SO_2_, CO, and NO_2_ gases.^19^ However, the selectivity properties of the reactive species are also what make them difficult to study with specificity and sensitivity.^20^ Since the selectivity of the reactions is largely determined by the chemical environment,^21^^,^^22^^,^^23^^,^^24^^,^^25^^,^^26^^,^^27^ the anion CHCl˙^–^ clusters could play a crucial role given the negative charge. For example, the buffer anion-like bicarbonate can promote a higher CO_2_ concentration compared to the nonbuffered system.^28^^,^^29^ Similarly, the halide anion (Cl^−^, Br^−^, and I^−^) clusters can partially block or modify active-site electronic structures, facilitating or suppressing specific reaction pathways.^30^ Of course, the intrinsic molecular structure and polarity also contribute to its chemical selectivity. With those backgrounds, the present study focuses on eight clusters formed between the CHCl˙^–^ and oxygen-containing gases: O_2_ (Linear, nonpolar), CO (Linear, weak polar), CO_2_ (Linear, nonpolar), NO (Linear, weakly polar), N_2_O (Linear, weakly polar), NO_2_ (bent, moderately polar), COS (Linear, weakly polar), and SO_2_ (bent, strongly polar), on the basis of the multi-scale computational simulation strategy integrating MD simulations and DFT calculations. In this work, we establish a unified structure-dynamics-mechanism framework for gas-phase reactivity, employing MD to map the dynamic microenvironment and DFT to quantify the intrinsic energetics of elementary steps. The overall simulation workflow is illustrated in Figure 1 and the corresponding computational details are provided in the Computational Details section. As an important purpose, by highlighting the vital role of anion cluster selection in enabling a highly efficient reaction, this study offers valuable insights into the formation mechanisms of interstellar organic molecules, suggests potential applications in the treatment of chlorine-containing waste gases, and guides the rational design of novel chlorinated carbene-based reagents.

## Results and discussions

### MD simulations

**Figure 2:**
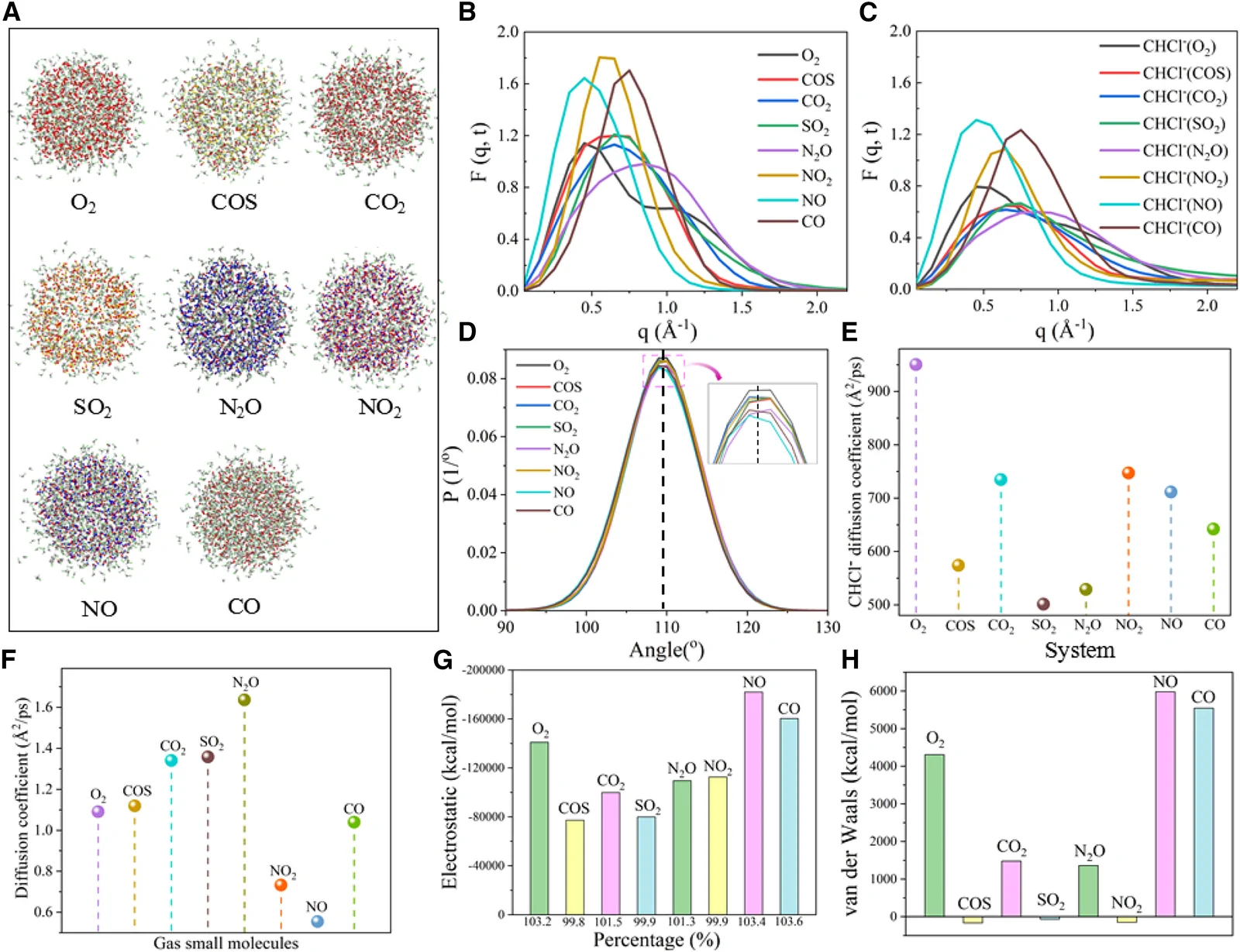
Clusters consist of the CHCl˙^–^ anion with eight oxygen-containing species (A) Equilibrium clusters. (B) Space time correlation function of gas molecules. (C) Space time correlation function of CHCl˙^–^ within various gases. (D) Bond angles change of CHCl within various gases. (E) CHCl˙^–^ diffusion coefficient. (F) Gas molecules diffusion coefficient. (G) Electrostatic energy and the corresponding contribution to total energy. (H) van der Waals energy.

**Figure 3:**
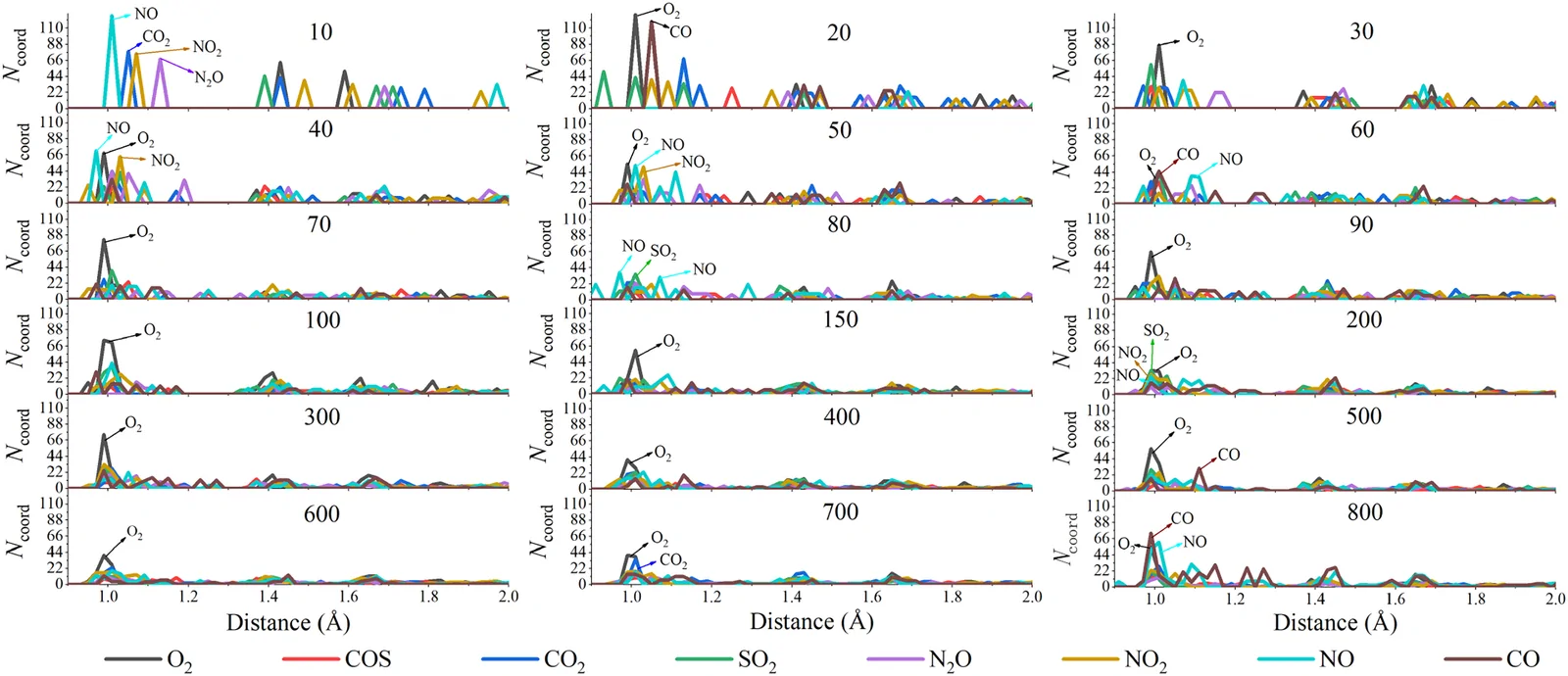
MD simulated the local coordination number (*N*_coord_) profiles of the eight gas small-molecules around the CHCl˙^–^ in the equilibrium system

**Figure 4:**
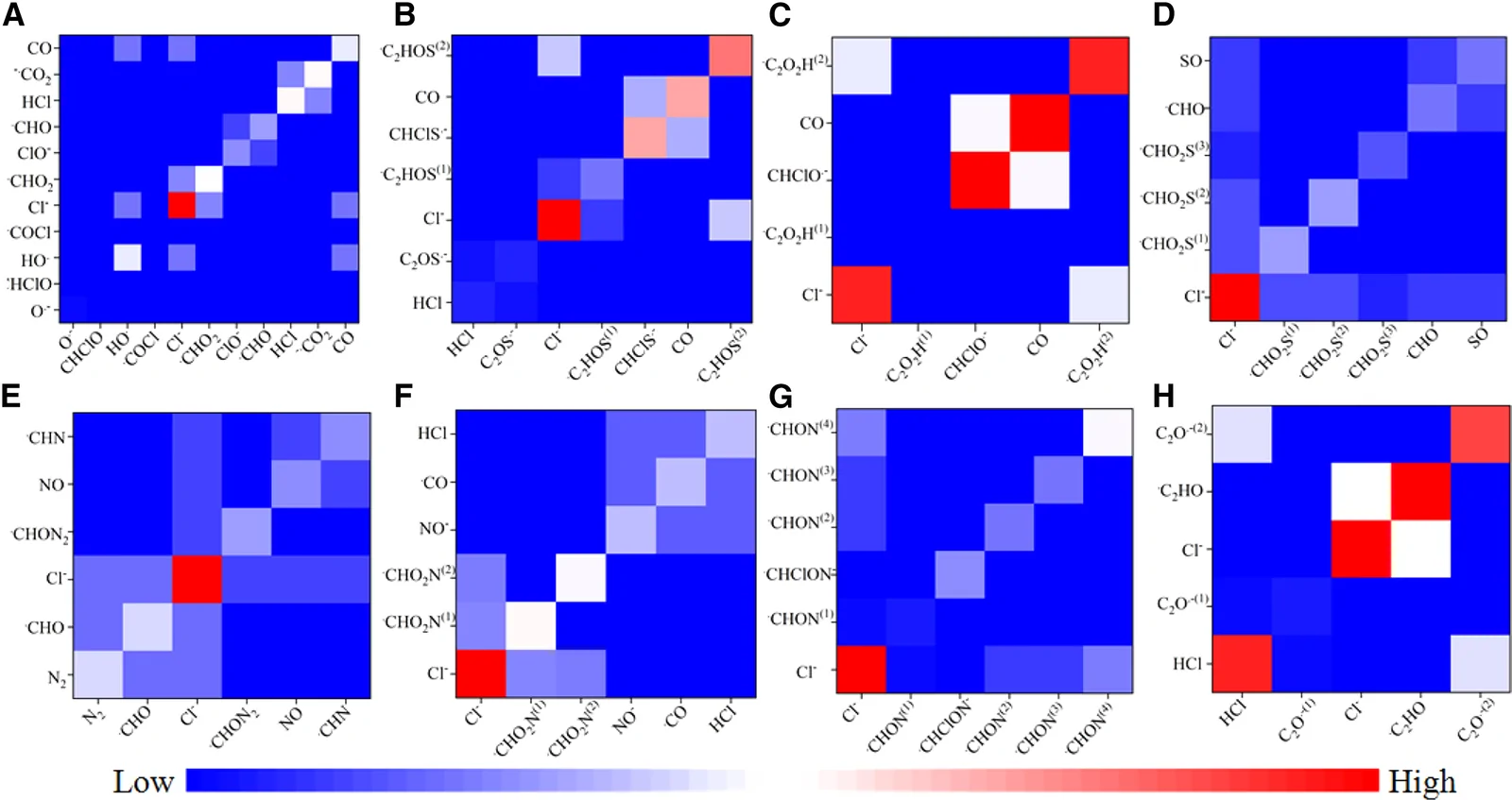
Correlation matrix of product species and their corresponding quantities from MD ReaxFF simulation For structural isomers, chemical formulas are also marked with superscripted parentheses containing Arabic numerals. (A) O_2_ reaction, (B) COS reaction, (C) CO_2_ reaction, (D) SO_2_ reaction, (E) N_2_O reaction, (F) NO_2_ reaction, (G) NO reaction, and (H) CO reaction. The protocol involved: (A) initializing the system with one CHCl˙^–^ and multiple gas molecules; (B) running NVT-MD at 300–500 K for 500 ps with a 0.25 fs time step; and (C) monitoring bond orders to identify bond cleavage and formation events.

After geometry optimization, followed by annealing, and then MD structural relaxation, the configurations of the gas-adsorbed-CHCl˙^–^ clusters exhibit a similar and consistent trend, indicating that the gas molecules tend to localize near the cluster center while the CHCl˙^–^ anion diffuses outward, as illustrated in Figure 2A. This arrangement allows a single CHCl˙^–^ to interact with multiple gas molecules, potentially enhancing its sensitivity toward oxygen-containing gases. This feature was also corroborated by the MD-derived F(q, t) data. As shown in Figures 2B and 2C, both the gas molecules and CHCl˙^–^ exhibit slow F(q, t) decay, indicating correlated, restricted translational and rotational dynamics driven by strong intermolecular interactions within the cluster. The pronounced slowing down is particularly evident for NO_2_, NO, and CO systems. Nevertheless, the propensity for chemical reactivity is ultimately governed by the electronic structure of the reactants, which is addressed in the following DFT study. In addition, among the eight cluster configurations examined, the one containing COS molecules exhibit a slightly flatter geometry compared to the other seven, which display approximately spherical packing. This deviation reflects an anisotropic molecular arrangement, likely arising from the asymmetric linear structure and weak polarity of the COS molecule. Structural analysis of the clusters (Figure 2D) reveals that the H–C–Cl bond angles in CHCl˙^–^ peak are predominantly distributed around 110° across all gas environments. The highest population occurs in the O_2_ system; the lowest in NO. This suggests CHCl˙^–^ may possess more activated sites in an O_2_-rich environment. Diffusion coefficient (*D*) calculations support this trend. As shown in Figure 2E, CHCl˙^–^ exhibits the fastest diffusivity in the O_2_ system (largest *D* value) and the slowest in the SO_2_ system—not NO as might be expected. This hindered mobility aligns with earlier reports attributing it to SO_2_ aggregation tendency.^31^ Interestingly, NO itself shows the lowest diffusivity among all gases (Figure 2F), which we attribute to its intrinsic stability and strong self-interaction. Conversely, N_2_O diffuses fastest due to its low molecular mass relative to SO_2_. Moreover, energy decomposition analysis (Figures 2G and 2H) indicates that electrostatics dominate over vdW contributions to the total interaction energy in all systems, supporting the role in overall stability of the clusters and validating the use of the COMPASS III force field. Notably, the vdW interactions make a stabilizing (negative) contribution only for the COS, SO_2_, and NO_2_ systems; in contrast, they are destabilizing (positive) for O_2_, CO_2_, N_2_O, NO, and especially CO systems. In the CO cluster, electrostatic energy accounts for approximately 103.6% of the total attractive interaction, implying a net vdW repulsion. To investigate gas selectivity, we varied the CHCl˙^–^ concentration (10–800 ions) in aerosol models containing 100 gas molecules each. The corresponding equilibrium configurations are provided in Figure S1. The *N*_*coord*_ provides a quantitative measure of the average number of gas molecules residing within the first solvation shell of the CHCl˙^–^ anions. The resulting *N*_*coord*_ profiles (Figure 3) reveal preliminary reactivity trends. Notably, O_2_ consistently associates strongly with CHCl˙^–^ across most concentrations, except at very low (10) and intermediate (80) anion counts, where the interaction appears weaker. In contrast, COS exhibits persistently weak RDF peaks regardless of CHCl˙^–^ concentration, indicating minimal affinity. Furthermore, reaction evolution simulations were conducted—based on the reactive force field (ReaxFF)—for trajectory configurations comprising 500 molecules of a single gas species interacting with 500 CHCl˙^–^ anions. These predict possible product species and their relative abundances. The corresponding correlation matrix was presented in Figure 4. In the reactions involving O_2_, SO_2_, N_2_O, NO_2_, and NO gases, the anion Cl^−^ was prominently yielded among the products. In contrast, the COS, CO_2_, and CO reactions produced Cl^−^ alongside significant ˙C_2_HOS, ˙C_2_O_2_H, and ˙C_2_HO radicals. Obviously, MD simulations can provide valuable insights into diffusion behavior and thermodynamic stability; however, they are inherently incapable of resolving electronic structure effects or chemical selectivity arising from the fundamental reaction mechanism. Therefore, DFT calculations were employed to uncover the origin of the observed selectivity, and the details will be elucidated as follows.

### DFT calculations

#### Reactants characteristics

**Figure 5:**
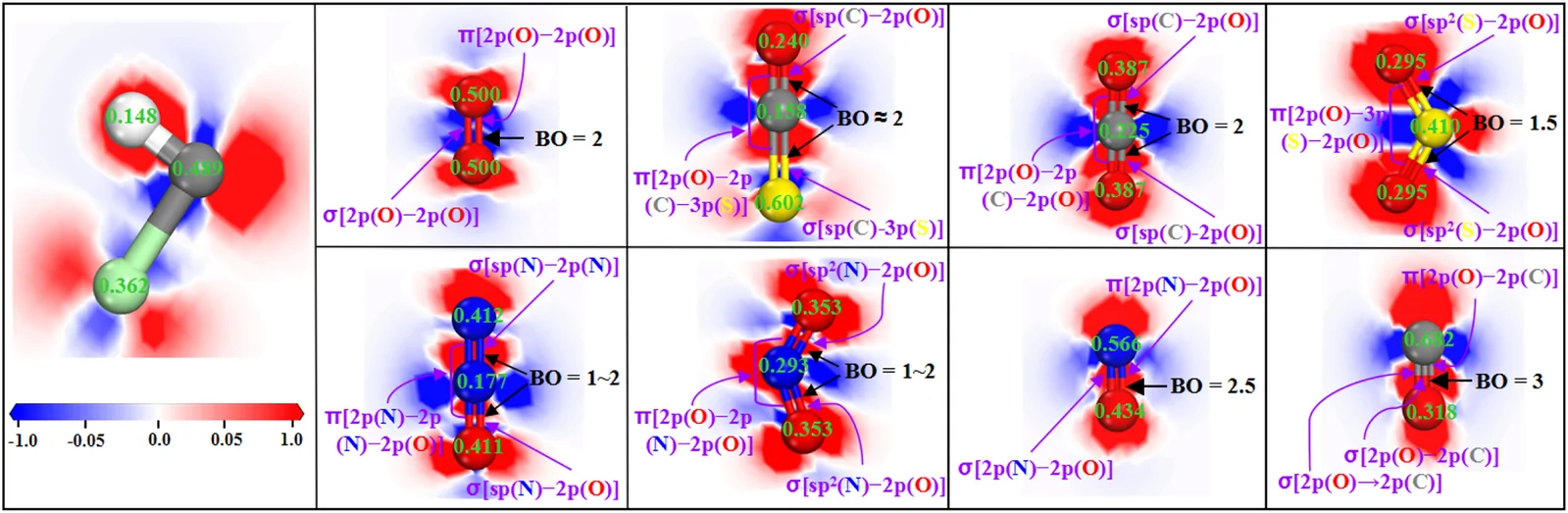
DFT computational bonding type and model, bond orders (BO), and electron density difference (EDD) of the reactant’s molecules together with the Fukui charges

In contrast to the other gases, which were treated as singlets, O_2_ was modeled in its triplet ground state (^3^Σ_g_^−^), consistent with its electronic configuration under ambient gas-phase conditions.^32^^,^^33^^,^^34^ It is very important to compare bond orders (BO) of these gas molecules to gain a deeper insight into their activity. As shown in Figure 5, the O_2_ molecule features one *σ* bond formed by overlap of the 2p_z_ orbital and two three-electron *π* bonds ($\pi_{2}^{3}$), which overall exhibit double bond characteristics with a bond order of 2. In the COS molecule, the central C-atom adopts sp hybridization and forms two *σ* bonds with the p-orbitals of the O and S atoms, respectively. The non-hybridized p orbitals on C, O, and S form two perpendicular π systems, giving rise to a cumulene-like O=C=S structure. For CO_2_, there are two *σ* bonds formed by each sp(C)-2p(O) overlap and two sets of delocalized $\pi_{3}^{4}$ bonds, yielding a bond order of 2. The SO_2_ molecule contains two localized *σ* bonds and a three-atom four-electron *π* bond ($\pi_{3}^{4}$), leading to an average S–O bond order of approximately 1.5. As the isoelectronic analog of CO_2_, the N_2_O molecule was characterized by two *σ* bonds formed via sp hybridized orbitals and two sets of delocalized *π* bonds ($\pi_{3}^{4}$). Consequently, both the N–N and N–O bonds display intermediate lengths between single and double bonds. The NO_2_ molecule is a species with an odd number of electrons, placing 3 N-atom sp^2^ hybrid orbitals, two of which form *σ* bonds, and one accommodates a single unpaired electron. Additionally, a delocalized $\pi_{3}^{4}$ bond contributes to partial double-bond character, resulting in N–O bond lengths that lie between those of single and double bonds. Similarly, NO, an open-shell molecule, has 8 bonding and 3 antibonding electrons, resulting in a final 2.5 BO. In contrast, as the isoelectronic species of the N_2_ molecule, the CO contains 14 valence electrons and features a triple bond, making it exceptionally stable. Clearly, from the BO perspective, the CO appears the least reactive among these species. However, chemical reactivity is not governed solely by bond strength; molecular polarity is also critical, as often reflected in specific chemical reactions. Therefore, a detailed mechanistic investigation is extremely necessary. Of course, the preferred reaction sites of these gas molecules were identified to be S atom (COS reaction), O atom (CO_2_ reaction), S atom (SO_2_ reaction), terminal-N and O atoms (N_2_O reaction), O atom (NO_2_ reaction), N atom (NO reaction), and C atom (CO reaction), respectively, by inspection of the corresponding Fukui charges values collected in Figure 5, according to the principle that the higher Fukui charge values correlate with the greater atomic reactivity. Meanwhile, due to carrying a relatively large Fukui charge, the C atom of CHCl˙^–^ serves as the primary nucleophilic center in initiating these reactions. However, Fukui charges only indicate inherent reactivity tendencies of the reactants. To assess actual charge redistribution, electron density difference (EDD) analysis is more informative. As shown in Figure 5, the EDD maps reveal significant electron accumulation on the C-atom of the CHCl˙^–^ anion acting as a nucleophile, while the oxygen-containing species exhibit the key electrophilic centers on their C and S atoms (COS), C atom (CO_2_), S atom (SO_2_), central-N atom (N_2_O), N atom (NO_2_), N atom (NO), and C atom (CO) because of the pronounced electron depletion. Considering donor-acceptor orbital interactions between the C (2p_z_) orbital of CHCl˙^–^ and the σ∗ orbitals of the gases, six distinct bond-cleavage pathways are possible. For clarity, we categorize these into four reaction types: (1) O-O bond activation, (2) C-O and C-S bond activation, (3) N-O and N-N bond activation, and (4) S-O bond activation. Further details will be discussed in subsequent sections.

#### O-O bond activation

**Figure 6:**
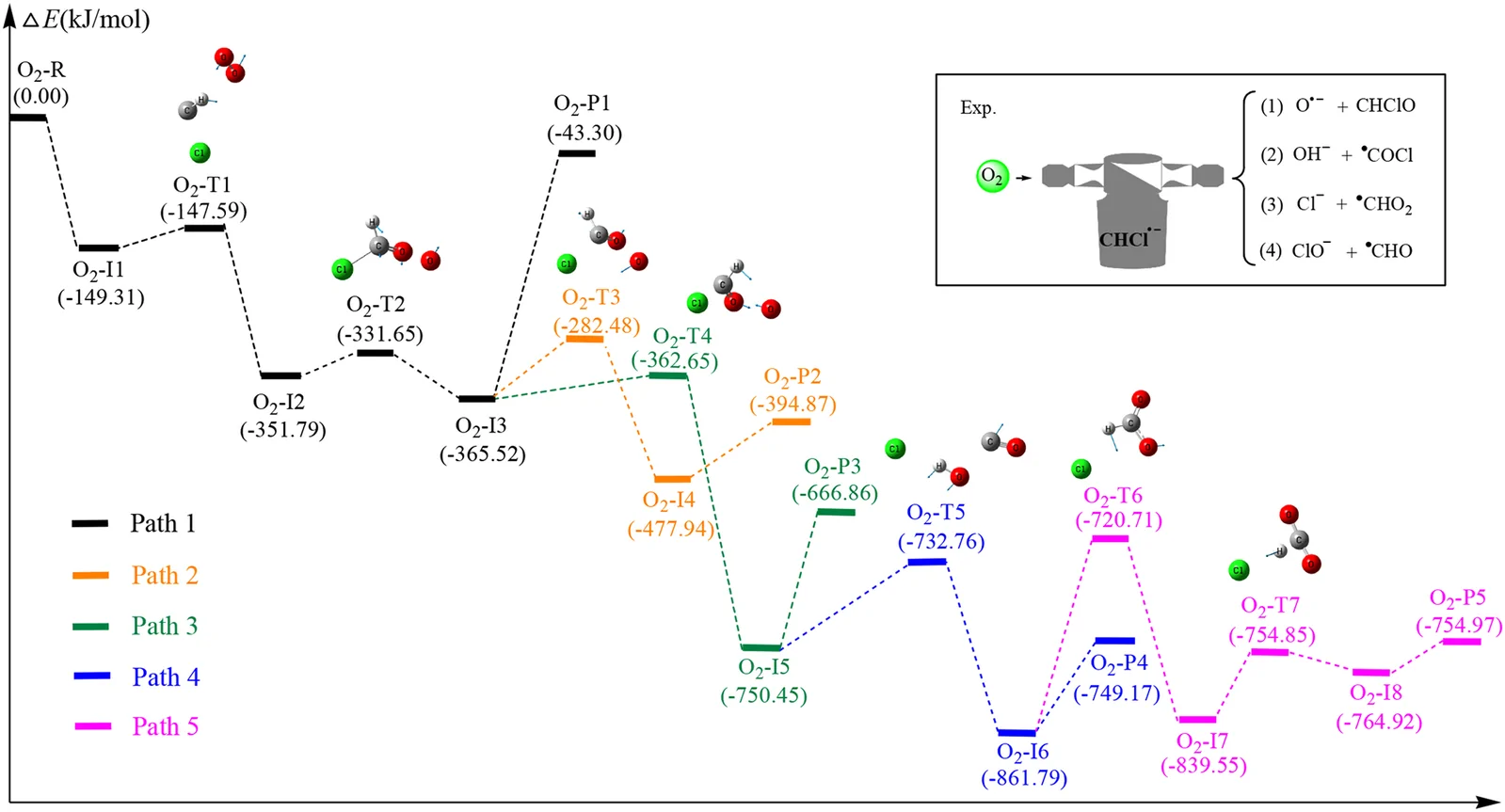
Energy profiles of the O_2_ reaction. Relative energies are taken from the QCISD(T)/aug-cc-pVDZ//BHandHLYP/aug-cc-pVDZ energies summarized in Table S2 Optimal mechanism for the O_2_ reaction: O_2_-R → O_2_-I1 → O_2_-T1 → O_2_-I2 → O_2_-T2 → O_2_-I3 → O_2_-T4 → O_2_-I5 → O_2_-T5 → O_2_-I6 → O_2_-P4.

The reaction of O_2_ with CHCl˙^–^ is chosen to investigate the O-O bond activation in this section. As outlined in Figure 6, the first pathway begins with O_2_-I1. It proceeds via transition state O_2_-T1 (29i cm^−1^) to undergo the activation of the O_2_, with a barrier of 1.72 kJ/mol. Such a lower barrier height can be attributed to the stronger electronegativity of O2. Obviously, after crossing O_2_-T1, which is labeled by a transition vector indicating the movement of O_2_ toward the C atom of the CHCl˙^–^, the intermediate O_2_-I2, whose O atom now links to the C atom, is easily generated with a significant exothermic reaction energy of −351.79 kJ/mol. As a result, the O_2_-I2 readily loses O˙^–^ radical. Indeed, due to the high activity, the O˙^–^ is difficult to exist alone; that is, the O˙^–^ can abstract either a Cl or H atom to form ClO^−^ or OH^−^ and the associated PES as a result of the O_2_-P2 or O_2_-P3 formation. As a prediction, beginning with O_2_-I2, the C-Cl bond is activated via O_2_-T2 (123i cm^−1^) with a barrier of 20.14 kJ/mol, yielding stable intermediate O_2_-I3 with complexation energy of −365.52 kJ/mol. Then, from the product-like complex O_2_-I3, the separated product O_2_-P1 (Cl^−^ + ˙CHO_2_) can be obtained. However, the O_2_-P1 formation is characterized by a very high barrier of 322.22 kJ/mol. In fact, it should be noted that the O_2_-P1 lies below the reactant energy level, as shown in Figure 6. Therefore, the anion Cl^−^ can be produced via the O_2_-I1→O_2_-T1→O_2_-I2→O_2_-T2→O_2_-I3→O_2_-P1 process, which is labeled Path 1 in Figure 6. Following that, the O_2_-reaction then continues from O_2_-I3 and divides into two distinct branches, one of which produces O_2_-P2 (Path 2) and the other O_2_-P3 (Path 3). As a comparison, the former (O_2_-P2: ClO^−^ + ˙OCH) needs to overcome an 83.04 kJ/mol barrier via O_2_-T3 with an energy release of −29.35 kJ/mol, whereas the latter (O_2_-P3: OH^−^ + ˙COCl) requires only 2.87 kJ/mol barrier crossing via O_2_-T4 with a massive reaction energy of −301.34 kJ/mol. Thus, from the thermodynamic and kinetic viewpoints, the product OH^−^ forms in a much easier way. This can be well understood by the instability of O˙^–^ in the reaction of O_2_ + CHCl˙^–^. From O_2_-I5, cleavage of a C-Cl bond with the help of the OH unit continues to occur via O_2_-T5 to generate a more stable intermediate O_2_-I6. This is referred to as Path 4 in this paper. Although this step requires overcoming an energy barrier of 17.69 kJ/mol, formation of stable O2-I6 is energetically favored with an overall exothermic energy of −861.79 kJ/mol, which is large enough to compensate and maintain a favorable profile. In this work, the overall exothermic energy is defined by the relative energy of a complex structure on a specific reaction pathway with respect to the zero-energy reference of the corresponding isolated reactants. In fact, because the O_2_-T5 is located well below the zero-energy level, the formation of O_2_-I6 is spontaneous without any additional activation energy. Thus, the predicted Cl^−^ formation via Path 4 is strongly favored over Path 1. Likewise, this also gives a better understanding of the significantly large abundance of Cl^−^ obtained from the reaction of O_2_ + CHCl˙^–^. Our MD simulations highlights a critical role for CHCl˙^–^ diffusion (Figure 2E), which displays the highest *D* value within the cluster. This allows CHCl˙^–^ to readily navigate the local environment to reach O_2_ gas, facilitating the low-energy pathways. Although the thermodynamic driving force for Cl^−^ formation via Path 4 is favorable with a substantial energy release of −749.17 kJ/mol, the reaction requires tight intermolecular contact between the reactants to overcome the rate-limiting C-Cl bond cleavage at the transition state O_2_-T5. The rapid diffusion of CHCl˙^–^ lowers the entropic barrier by ensuring frequent reactant collisions, thus explaining the prevalence of Cl^−^ observed experimentally. In addition, despite the fact that our QCISD(T)/BHandHLYP computed relative energy of O_2_-P4 (Path 4) is approximately −749.17 kJ/mol, when considering the consumption energy due to the compensation for formation of O_2_-P1 (Path 1), it matched well energy release values obtained from high-level G3 calculation (Δ*H* = ∼ −760.66 kJ/mol) and the experimental observations of Cl^−^ formation,^11^ also allowing us to further evaluate the appropriateness of our methods in the current study. Emanating from complex O_2_-I6 that can produce O2-P5 (HCl + ˙CO2−2–), the pathway is defined as Path 5, and its rate-limiting step is via O_2_-T6. However, the rate-limiting step of this pathway faces a much higher energy barrier of 141.08 kJ/mol, especially due to the exceptional stability of O2-I6, which is located on the PES with a complexation energy of −861.79 kJ/mol. Thus, the HCl production should be less likely in theory, despite the fact that the two transition states for the pathways, O_2_-T6 and O_2_-T7, are below the O_2_ + CHCl˙^–^ asymptote.

#### C-O and C-S bond activation

**Figure 7:**
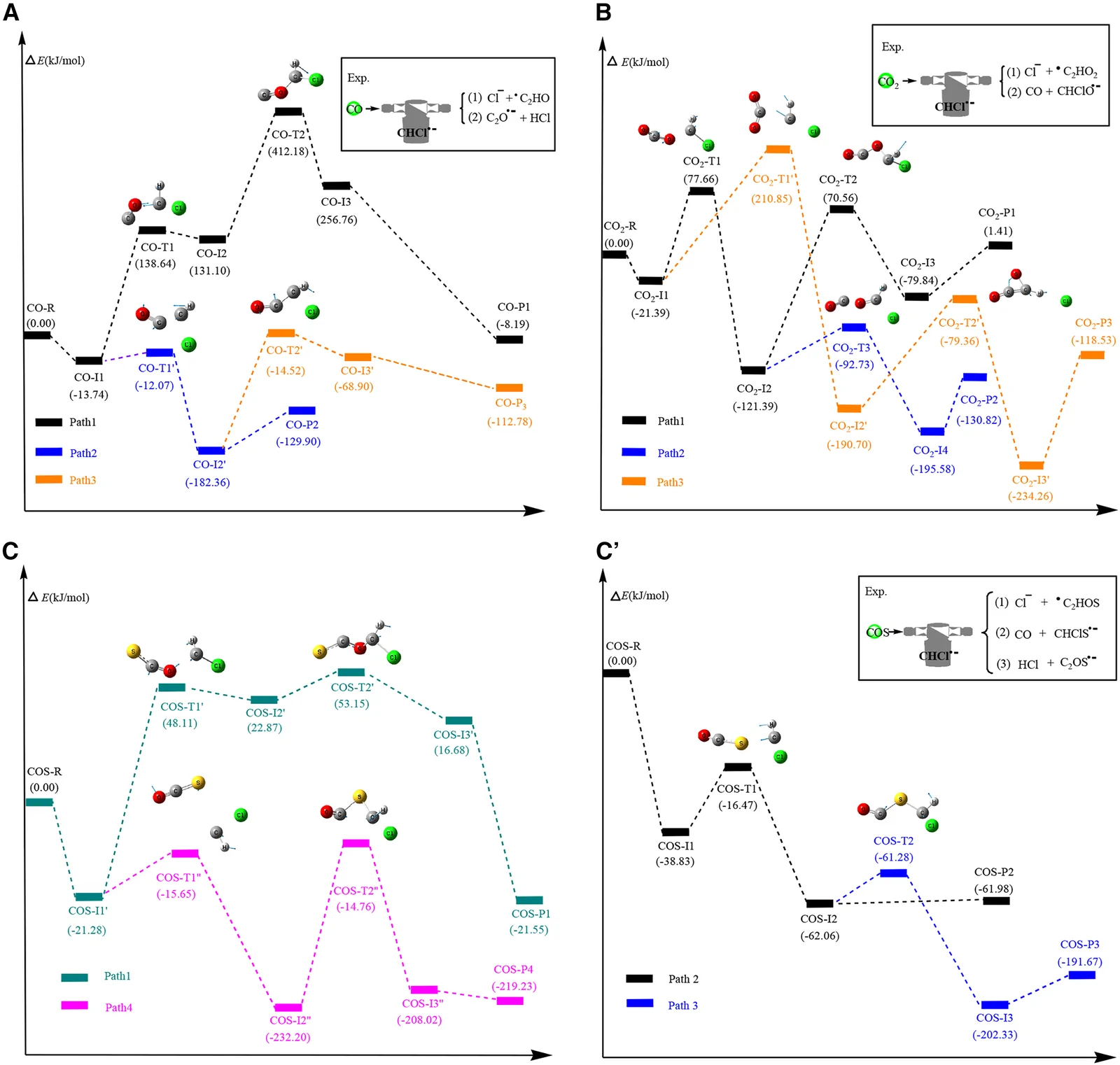
Energy profiles for the CO, CO2, and COS reactions Relative energies are reported at the QCISD(T)/aug-cc-pVDZ//BHandHLYP/aug-cc-pVDZ level and are summarized in Table S2 (supplemental information). (A) Reaction of CO. The optimal mechanism proceeds via CO-R → CO-I1 → CO-T1′ → CO-I2′ → CO-P2. (B) Reaction of CO2. The optimal mechanism proceeds via CO2-R → CO2-I1 → CO2-T1 → CO2-I2 → CO2-T3 → CO2-I4 → CO2-P2. (C/C′) Reaction of COS. The optimal mechanism proceeds via COS-R → COS-I1 → COS-T1 → COS-I2 → COS-T2 → COS-I3 → COS-P3.

The schematic PES profiles associated with possible C-O bond activation pathways derived from the reactions of CO, CO_2_, and COS are plotted in Figure 7, with Figure 7C′ specifically displaying the COS C-S bond activation. As demonstrated in Figure 7, the initial step in all pathways is the formation of the ion-molecule complexes, CO-I1, CO_2_-I1, COS-I1′, and COS-I1, with releasing of −13.74 (CO), −21.39 (CO_2_), −21.28 (COS: C-O bond activation), and −38.83 (COS: C-S bond activation) kJ/mol, respectively. These strongly imply that the complexes can be obtained without the use of additional energy. Following that, two distinct reaction modes for the C-O bond dissociation are discovered, demonstrating the C-attack and the O-attack, whereas only one pattern, the S-attack, is identified for the C-S bond rupture. The C-attack, O-attack, and S-attack were defined here as the C-atom, O-atom, and S-atom of the studied gas species interacting with CHCl˙^–^, respectively. This is not analogous to the insertion-elimination of CHCl˙^–^ mechanism in the halomethane reactions, presumably due to the relatively large C-Cl bond polarity.^6^ Of the three studied reactions, only the CO2 reaction exhibits two high-energy transition states, one for the O-attack (CO_2_-T1) and the other for the C-attack (CO_2_-T1′). Both lie above the reactant asymptote (Figure 7B). Thus, from a kinetic standpoint, the pathways of CO_2_ C-O bond activation by CHCl˙^–^ should be difficult to track down. This appears to contradict Villano et al.’ experimental observations,^11^ which predicted the survey of CO_2_ + CHCl˙^–^ → Cl^−^ + ˙C_2_HO_2_ and CO + CHClO˙^–^. MD simulations reveal that CO_2_ possesses only moderate diffusivity; however, the delocalization of its electrostatic potential over both oxygen atoms diminishes site-specific electrophilicity. Consequently, even upon occasional encounters with the CHCl˙^–^ anion, the energy barrier for C-O bond activation remains prohibitively high under ambient conditions. According to the gas-phase experiment at low pressure, which facilitates significant translational-to-vibrational energy transfer during collisions,^35^ we predict that the CO2 + CHCl˙^–^ reaction will take place at a higher temperature than room temperature. It should be noted that our calculated energies of the final products relative to reactants are (kJ/mol) +1.41 (CO_2_-P1: Cl^−^ + ˙C_2_HO_2_ with ternary ring), −130.82 (CO_2_-P2: CO + CHClO˙^–^), and −118.53 (CO_2_-P3: Cl^−^ + ˙C_2_HO_2_), indicating that CO formation is preferable to Cl^−^ ion formation. However, the elaborated experiments for the CO_2_ + CHCl˙^–^ reaction report that branching ratios (BR) of Cl^−^ and CO are 94% and 6%, respectively. One important reason for such an appearance is that the reaction is taking place at ∼302 K, where the reaction conditions make the CHCl˙^–^ more nucleophilic, thereby promoting attack on the electrophilic carbon of CO_2_. This favors formation of the CO_2_-P3 product. The characteristics parallel the reaction of CO_2_ with amines to form carbamates.^36^ As a result, the thermal stability of the product increases via bonding with reactive ions, which can improve CO_2_ selectivity.

Unlike the CO_2_ reaction, which favors the O-attack over the C-attack, both COS- and CO-reactions preferentially undergo C-attack for C-O bond cleavage, demonstrating the interaction energy for the C-attack are significantly larger than for the O-attack (cf. Figure 7). Meanwhile, for the COS reaction, the S-attack pathway is also accessible, as its associated complexes are all located below the zero-energy level as depicted in Figure 7C′. Therefore, the HCl production should be less from COS + CHCl˙^–^ or CO + CHCl˙^–^ reactions. As expected, the BR of HCl formation was only ∼3% in the COS reaction and trace in the CO reaction. As a result of this encouraging agreement, the employed BHandHLYP functional was further proven as an adequate theoretical level for studying the title reactions. Moreover, as a comparison, the PES profiles discovered for C-O bond activation via the C-attack pathways for the COS and CO reactions driven by CHCl˙^–^ are very similar. As demonstrated in Figure 7, a C-C bonding complex can be easily formed via an initial transition state from the ion-molecule complexes, with energy barriers of 1.67 kJ/mol in the CO reaction (CO-I1→CO-T1′→CO-I2′) and 5.63 kJ/mol in the COS reaction (COS-I1″→COS-T1″→COS-I2″). Of course, relatively high barrier in the COS reaction that can be explained by the existence of an alternant reaction pattern, the S-attack, which is fiercely competitive with the C-attack. However, CHCl˙^–^ preferentially attacks the carbon atom of COS rather than sulfur. This C-C interaction significantly accelerates the COS reaction, establishing C-attack as the primary source of the experimentally detected Cl^−^ ions, despite the S-attack being energetically favored. With respect to the formed C-C connection complex, the COS-I2″ (in COS reaction) is 49.84 kJ/mol more stable than the CO-I2′ (in CO reaction), highlighting that the CHCl˙^–^ can be the better candidate to react with the COS than the CO. Noted that, starting from the CO-I2′ complex, one hand, the CO reaction can directly and readily yield Cl^−^ + ˙C_2_HO (CO-P2) with an exothermic energy of −129.90 kJ/mol. On the other hand, it will continue to proceed, as in Path 3, through the H-migration transition state CO-T2′ to form HCl + C2O˙^–^ products (CO-P3) with a 167.84 kJ/mol energy barrier, implying a trace HCl yield. Similarly, the COS reaction must overcome a high barrier of 217.44 kJ/mol via COS-T2″ to produce the final product COS-P4 (Cl^−^ + ˙OC_2_HS) starting from the complex COS-I2″. Therefore, although the barriers to the generation of the CO-P3 and COS-P4 are quite high, they may still be overcome because the corresponding TSs lie below the reactant energy level, as a result of trace HCl detected in the experiments of the COS and CO reactions. MD simulations illuminate the underlying physical constraints. For COS, a flattened geometry and weak polarity hinder solvation and C=S bond polarization, eliminating both thermodynamic and kinetic viability. Conversely, CO is hampered by its extreme C-O triple bond strength and slight vdW repulsion, which create insurmountable kinetic barriers despite favorable electrostatic contacts. Thus, while COS lacks thermodynamic incentive, CO is immobilized by its intrinsic bond strength.

#### N-O and N-N bond activation

**Figure 8:**
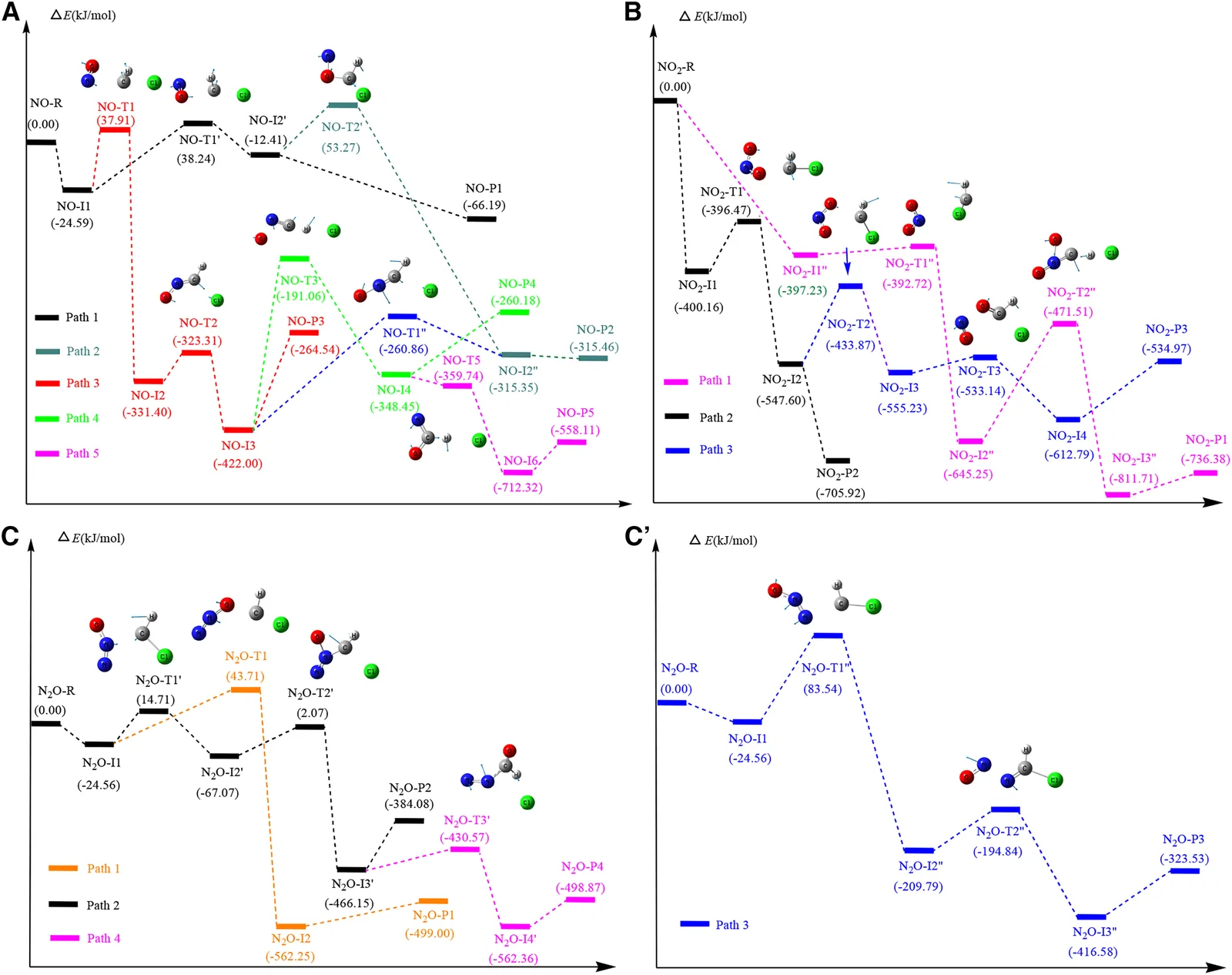
Energy profiles for the NO, NO2, and N2O reactions Relative energies are reported at the QCISD(T)/aug-cc-pVDZ//BHandHLYP/aug-cc-pVDZ level and are summarized in Table S2 (supplemental information). (A) Reaction of NO. The optimal mechanism proceeds via NO-R → NO-I1 → NO-T1 → NO-I2→ NO-T2 → NO-I3 → NO-P3. (B) Reaction of NO2. The optimal mechanism proceeds via NO2-R → NO2-I1 → NO2-T1 → NO2-I2 → NO2-P2. (C/C′) Reaction of N2O. The optimal mechanism proceeds via N2O-R → N2O-I1 → N2O-T1′ → N2O-I2′ → N2O-T2′ → N2O-I3′ → N2O-T3′ → N2O-I4′ → N2O-P4.

In this section, the reactions of NO, NO_2_, and N_2_O were chosen to examine the N-O bond activation. When CHCl˙^–^ encounters the nitrogen-containing molecules, a type of weakly bound complex is formed at first, with only one initial complex forming in the NO and N_2_O reactions and two initial isomers appearing in the NO_2_ reaction. Our calculations suggest these initial complexes are more reactant-like, and their formation can be spontaneous without adding extra energy. For the NO reaction, two pathways start from the same precursor NO-I1 and proceed via transition states, NO-T1 and NO-T1′, with energy barriers of 62.50 and 62.83 kJ/mol, respectively. Such a comparable difference in barriers can be explained by the similar electronegativity between the O and N atoms. However, the NO reaction via NO-T1 is more advantageous because the interaction energy for forming NO-I2 is approximately 30 times greater than that for forming NO-I2′. Therefore, attachment of the N atom of the NO molecule to C of the CHCl˙^–^ is strongly preferred. This aligns with the NO sensing experiment,^19^ which indicates that the oxygen atom does not interfere with NO detection. The kinetic insights from our MD simulations show that, as provided in Figure 2F, among the tested gases, NO exhibits the lowest diffusivity and significant aggregation tendencies. Even though NO possesses electrophilic nitrogen sites, its restricted mobility likely imposes a significant entropic penalty on reaction initiation, potentially hindering its reactivity despite favorable electronic interactions. Therefore, both dynamic accessibility and electronic structure govern the overall selectivity in these complex cluster environments. Coming from NO-I2 to NO-I3 via NO-T2, as shown in Figure 8A, the PES profile illustrates that the intermediate NO-I3 easily forms with a barrier of 8.09 kJ/mol. Next, three subsequent pathways associated with NO-I3 take place, (1) the pathway (Path 3) directly yielding product NO-P3 (Cl^−^ + ˙ONCH) with barrierless relative to the separated reactants, (2) the pathway (Path 6) involving NO-T1″ to give complex NO-I2″, characterized by the energy barrier of 161.14 kJ/mol, and (3) the pathway (Path 4) involving the isomerization of the ONCH group from the linear (NO-I3) to the non-linear (NO-I6) via NO-T3 and NO-T5 in turn, with a rate determining barrier of 230.94 kJ/mol. Indeed, all stationary points on the PES for the three discovered pathways are located below the zero-energy reference, allowing those pathways to be achieved without the requirement of extra energy, as displayed in Figure 8A, thereby rendering Cl^−^ the predominant product.

PES profiles for the NO_2_-reaction are plotted in Figure 8B. As can be seen in Figure 8B, the CHCl˙^–^ + NO_2_ reaction begins with two distinct precursor complexes, NO_2_-I1 and NO_2_-I1″, which exhibit substantial interaction energies of −400.16 and −397.23 kJ/mol, respectively. The pathway starting from the former was defined as O-attack, while the pathway starting from the latter was referred to as N-attack. Both pathways face negligible kinetic barriers. The O-attack proceeds via NO_2_-T1 to form NO_2_-I2 with a 3.69 kJ/mol energy barrier, while the N-attack proceeds via NO_2_-T1″ to form NO_2_-I2″ with a barrier of 4.51 kJ/mol. Following that, for the O-attack pathway originating from NO_2_-I2, a barrierless channel yields NO_2_-P2 (Cl^−^ + ˙ONOCH). This exit path is flat and highly exothermic, by −705.92 kJ/mol, facilitating easy Cl^−^ formation. Meanwhile, the NO_2_-I2 continues to proceed via another pathway (Path 3) consisting of NO_2_-I2→NO_2_-T2→NO_2_-I3→NO_2_-T3→NO_2_-I4→NO_2_-P3 as given in Figure 8B, in which two distinct transition states, NO_2_-T2 and NO_2_-T3, are observed with the respective energy barriers of 113.73 and 22.09 kJ/mol. Clearly, the rate-determining step is the step from *trans*-configuration NO_2_-I2 to *cis*-configuration NO_2_-I3 via NO_2_-T2. Despite the high energy barrier in this step, the NO_2_-T2 is located beneath the zero-energy level. Accordingly, the Path 3 route, which produces the product NO_2_-P3 (NO^−^ + ˙CO + HCl), occurs more easily as well. Alternatively, the NO_2_-I2″ undergoes an intramolecular S_N_2-type displacement via the NO_2_-T2″. In this step, the oxygen atom attacks the electrophilic carbon of CHCl˙^–^ to displace Cl^−^, forming the complex NO_2_-I3″. However, this step requires a 173.74 kJ/mol high energy barrier to surmount, the intermediate NO_2_-I2″ in the stage is energetically stable, with a total exothermic energy of −645.25 kJ/mol. Indeed, as shown in Figure 8B, the NO_2_-T2″ resides well below the zero-energy level. Consequently, the intermediate NO_2_-I3″ forms spontaneously, making the production of NO_2_-P1 (Cl^−^ + ˙CHONO) thermodynamically feasible. Unlike NO, which is hindered by self-aggregation, NO_2_ benefits from a balanced diffusion profile and an open-shell electronic structure. The presence of an unpaired electron facilitates electron transfer and the formation of nitrite-like intermediates. Although the N-O bond order is fractional (∼1.5), this open-shell nature lowers the barrier for bond cleavage compared to closed-shell molecules like CO_2_. Our ReaxFF simulations corroborate this, showing NO_2_ readily initiates radical cascades leading to ˙C_2_O_2_H and Cl^−^ formation.

In comparison to the NO_2_ reaction, as demonstrated in Figure 8C/(C)′, the N_2_O reaction has three potential routes and initiates from the N_2_O-I1 complex, each characterized by a unique transition state, N_2_O-T1″, N_2_O-T1, and N_2_O-T1′. PES profiles show that the three TSs starting from the N_2_O-I1 give the energy barriers of 108.10, 68.27, and 39.27 kJ/mol, respectively. In addition, similar to the NO and CO_2_ reactions, all three TSs reside above the zero-energy reference level, contrasting with the behavior observed for the NO_2_ reaction. In fact, even though these pathways have a significant energy barrier, the N_2_O reaction is energetically feasible to get the intermediates N_2_O-I2″, N_2_O-I2, and N_2_O-I2′, respectively, since the reactions may be carried out at a higher temperature rather than at room temperature.^11^ In the next step, the yielded N_2_O-I2 then gives rise to the exit route, which leads to the formation of the product N_2_O-P1 (N_2_ + ˙OCH + Cl^−^). In comparison, an intramolecular S_N_2 pathway from N_2_O-I2′ via N_2_O-T2′ is now going on to form N_2_O-I3′, in which the oxygen atom attacks the carbon atom to displace Cl^−^ with a substantial exothermicity of −466.15 kJ/mol. Meanwhile, an N-N bond cleavage pathway from N_2_O-I2″ via N_2_O-T2″ is also evident (Figure 8C′). Of the three discussed transition states (N_2_O-T2′, N_2_O-T3′, and N_2_O-T2″), the N_2_O-T2′ induces the highest barrier of 69.14 kJ/mol, attributed to the appearance of a strained three-membered ring. By comparison, the C- N bond-breaking pathway in the N2O-I3′→N2O-T3′→N2O-I4′ step requires a 35.58 kJ/mol barrier; the N-N bond dissociation via N2O-T2″ is kinetically favored due to its lower energy barrier of 14.95 kJ/mol. However, analysis of the reaction energy profile indicates that the rate-determining step of the C-N bond cleavage for ultimate product N_2_ formation is the N_2_O-I2′→N_2_O-T2′→N_2_O-I3′ step with 69.14 kJ/mol barrier height, whereas the rate-determining step of the N-N bond dissociation for final product NO formation is the initial step N_2_O-I1→N_2_O-T1″→N_2_O-I2″ with 108.19 kJ/mol barrier. Because the higher energy barriers correlate with slower reaction rates, NO formation in the N2O reaction is actually less likely. This computational prediction aligns with the experimental observation,^37^ proposing that while N-N scission is plasma accessible, O-N cleavage remains the dominant decomposition pathway. Despite possessing a favorable electronic structure and exhibiting the highest self-diffusivity among the studied gases (Figure 2F), N_2_O exhibits limited reactivity within CHCl˙^–^ clusters. This kinetic inhibition, rooted in an intrinsic resistance to bond activation rather than mass transport limitations, negates its electronic advantages. These findings reveal that cluster reactivity is governed by a delicate interplay between dynamics and thermodynamics: high intrinsic reactivity is irrelevant without sufficient kinetic accessibility, and high mobility is inconsequential in the absence of thermodynamic driving forces. Only when both criteria are satisfied—as with O_2_—can a pathway achieve dominant feasibility. Our computational results are in good accord with available experimental data for CHCl˙^–^ ion–molecule reactions. Villano et al.^11^ reported rate constants (*k*, cm^3^ molecule^−1^ s^−1^) for CHCl˙^–^ with O_2_ (2.0 × 10^−9^), N_2_O (2.3 × 10^−10^), CO_2_ (<1 × 10^−12^), and CO (no reaction) using a flowing-afterglow selected-ion flow tube (FA-SIFT). They concluded that reactions with CO_2_ are thermodynamically disfavored while those with O_2_ proceed efficiently—fully consistent with our calculated reaction enthalpies showing CO_2_, NO, and N_2_O require elevated temperatures to overcome endothermic barriers. Moreover, the kinetic implications of the computed energy barriers were further evaluated using conventional transition state theory. Pathways with activation barriers exceeding ∼125 kJ/mol are predicted to have negligible rates (*k* < 10^–15^ s^−1^) under the investigated conditions 298–400 K, rendering them kinetically inaccessible despite potential thermodynamic favorability.^38^^,^^39^ Consequently, the experimentally observed product distribution is dictated solely by pathways with lower barriers (<105 kJ/mol). This kinetic filtering explains why certain thermodynamically viable channels—particularly those involving CO_2_, NO, and N_2_O activation—require elevated temperatures to proceed, as their higher barriers become surmountable only with increased thermal energy. Although entropic effects moderately influence the reaction free energies, the qualitative trends in reactivity and the necessity of elevated temperatures for activating CO_2_, NO, and N_2_O remain robust.

#### S-O bond activation

**Figure 9:**
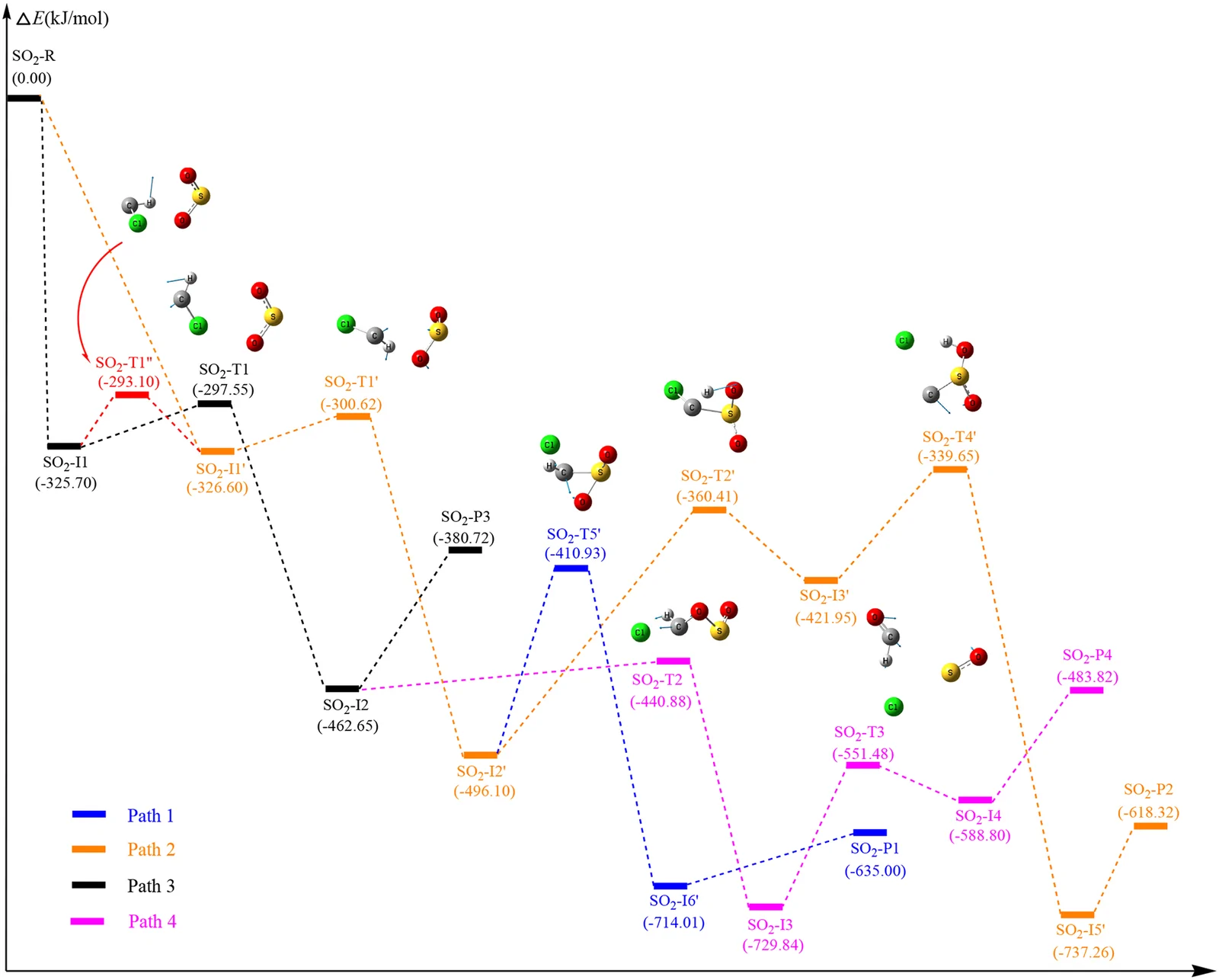
Energy profiles of the SO_2_ reaction Relative energies are taken from the QCISD(T)/aug-cc-pVDZ//BHandHLYP/aug-cc-pVDZ energies summarized in Table S2. Optimal mechanism for the SO_2_ reaction: SO_2_-R → SO_2_-I1 → SO_2_-T1 → SO_2_-I2 → SO_2_-T2 → SO_2_-I3 → SO_2_-T3 → SO_2_-I4 → SO_2_-P4.

With the assistance of CHCl˙^–^, the S-O bond activation of the SO_2_ molecule first yields two reactant-like weakly bonded complexes, SO_2_-I1 and SO_2_-I1′, and their formation is barrierless with significant exothermicity, as demonstrated in Figure 9. The PES profile indicates that an isomerization takes place from SO_2_-I1 to SO_2_-I1′ via SO_2_-T1″ with a barrier of 32.60 kJ/mol. However, the two complexes are quite close in energy, with the SO_2_-I1′ lying only ∼1 kJ/mol below the SO_2_-I1, suggesting that both species are likely to coexist under the investigated conditions. Starting from SO_2_-I1, the pathway named by Path 3 via SO_2_-T1 undergoes a C- O bond formation to yield the SO_2_-I2 complex with an energy release of −462.65 kJ/mol, in which the C atom of the CHCl unit bridges to one O atom of the SO_2_ segment. Note that this step has a 4.45 kJ/mol lower barrier than the isomerization step (SO_2_-I1→SO_2_-T1″→SO_2_-I1′), thus further validating SO_2_-I1′ is unlikely to form via the isomerization process. As a result, the SO_2_-I2 may readily be created as a product-like complex. The SO_2_-I2 then undergoes Cl^−^ ion detachment to produce SO_2_-P3 (Cl^−^ + ˙OCSOH) across a high barrier of 81.93 kJ/mol. Although the barrier is quite high, the overall formation of SO_2_-P3 remains thermodynamically favorable owing to the large exothermicity of −380.72 kJ/mol relative to the separated reactants. Alternatively, from the SO_2_-I2, another step (Path 4) can occur, characterized by the formation of a C-S bond, to generate intermediate SO_2_-I3. As shown in Figure 9, formation of the SO_2_-I3 requires a 21.77 kJ/mol barrier via SO_2_-T2 with an energy release of −729.84 kJ/mol. This behavior closely parallels that observed in the CF^+^ + SO_2_ reaction, where the branching ratio of the oxygen transfer product FCO^+^ is about 0.65.^40^ After that, beginning from the SO_2_-I3, the created C-S bond now breaks via SO_2_-T3 to yield the product-like complex SO_2_-I4. However, in order to produce SO_2_-I4, a barrier of 178.36 kJ/mol must be overcome. Clearly, the marked increase in barrier height of SO_2_-T3 compared to SO_2_-T2 implies that the SO_2_-I3→SO_2_-I4 conversion is kinetically disfavored, thereby making the formation of SO_2_-P4 less competitive. Nevertheless, since the SO_2_-T3 has a lower energy level than the separated reactants, the Cl^−^ ion product from SO_2_-P4 may still be experimentally accessible.

Another alternative S-O bond activation pathway also starts from the SO_2_-I1′ and proceeds via SO_2_-T1′ to lead to the formation of complex SO_2_-I2′, in which the S atom of SO_2_ is bridged to the C atom of CHCl˙^–^. Compared with SO_2_-I2 formation, the SO_2_-I2′ appears in a much easier way, requiring only a 25.98 kJ/mol barrier with respect to the SO_2_-I1′ with an energy release of −496.10 kJ/mol. Following that, two distinct reaction channels would occur from the SO_2_-I2′ (Figure 9), exhibiting SO_2_-I2′ → SO_2_-T5′ → SO_2_-I6′ → SO_2_-P1 (Path 1) and SO_2_-I2′ → SO_2_-T2′ → SO_2_-I3′ → SO_2_-T4′ → SO_2_-I5′ → SO_2_-P3 (Path 2), respectively. The former corresponds to an intramolecular S_N_2-type process, in which the oxygen atom attacks the carbon atom to displace Cl^−^. The step is characterized by a high barrier of 85.17 kJ/mol, presumably reflecting the geometric strain associated with the three-membered ring in the SO_2_-T5′ structure (cf. Figure 9). However, as compared to the separated reactants, the SO_2_-T5′ is placed below the zero-energy level; in particular, the creation of the product-like complex SO_2_-I6′ has a significant exothermicity of −714.01 kJ/mol. Thus, Path 1 is very favorable in thermodynamics. Of course, the substantial internal energy accumulated in SO_2_-I6′ readily promotes further evolution toward SO_2_-P1 (Cl^−^ + ˙OCHSO), accounting for the prominent appearance of the Cl^−^ ion in the final product distribution. By comparison, Path 2 can also yield the Cl^−^ ion after passing through SO_2_-T2′ (H-transfer) followed by SO_2_-T4′ (Cl^−^ release). Nevertheless, because of a 50.52 kJ/mol higher barrier characterized for the Path 2 rate-determining step (SO_2_-I2′→SO_2_-T2′→SO_2_-I3′) compared to that of the intramolecular S_N_2 pathway found in Path 1, Path 2 is kinetically disfavored despite the SO_2_-I5′ formation induce a considerable exothermicity of −737.26 kJ/mol. In contrast to the NO case, these results highlight that for SO2, a strong thermodynamic driving force can effectively compensate for inherently sluggish kinetics. Although CHCl˙^–^ exhibits the most restricted diffusivity, tending to aggregate within the SO_2_ environment (Figure 2E), observable reactivity persists, consistent with the electrophilic character of SO_2_ as corroborated by Fukui charge and EDD analyses (Figure 5). Once diffusive constraints are overcome, the S-O bond activation becomes strongly exothermic. Overall, SO_2_ occupies an intermediate position in reactivity, displaying less kinetically competitive than O_2_ but more reactive than COS.

### Conclusions

This study, through multi-scale simulations of CHCl˙^–^ reactions, elucidates the molecular reaction mechanisms underlying its interactions with a series of gaseous oxidants. The following are the key conclusions from our simulations and computations. MD simulations reveal that CHCl˙^–^ diffuses outward and then interacts with various tested gas molecules, exhibiting that CO acts as a kinetic fortress, NO behaves as a diffusive prisoner, SO_2_ emerges as a thermodynamic survivor, and only O_2_ stands out as a kinetic-thermodynamic champion. DFT calculations demonstrate that the gases O_2_, NO_2_, SO_2_, and CO is activated effectively by CHCl˙^–^, with overall reactivity decreasing in the order O_2_ > NO_2_ > SO_2_ > CO. In all cases, the most energetically favorable pathways arise when the C atom of CHCl˙^–^ interacts with any atoms within the target gas molecules. Notably, in the case of CO, the dominant channel proceeds via C-C coupling rather than O-C interaction. The reactivity toward COS is governed by competitive attack at the C or S atom, yielding overall weaker activity compared to its structural analog SO_2_ and favoring Cl^−^ as the primary anionic product. For CO_2_, NO, and N_2_O reactions, the transition states of their rate-determining steps lie above the zero-point energy reference, implying that elevated temperatures are required to drive these reactions—aligning well with reported experimental behavior.

By bridging molecular-level dynamics with electronic-structure insights, our results demonstrate that gas-phase anion-molecule reactivity cannot be rationalized by bond energy or charge distribution alone. Instead, it emerges from the convoluted interplay of diffusion kinetics, intermolecular compatibility, and intramolecular lability. Only when these factors align does a reaction pathway become both kinetically accessible and thermodynamically favored. This work provides a predictive foundation for radical-anion chemistry, opening new ways for addressing complex challenges in gas-phase chemistry.

### Limitations of the study

While this work provides molecular-level insights into the reactivity of CHCl˙^–^ anions, several limitations should be acknowledged. First, the computational models primarily focus on idealized cluster environments; therefore, the influence of extended solvation effects, external electric fields, or heterogeneous surfaces prevalent in realistic interstellar or plasma environments is not explicitly captured. Second, although temperature-dependent thermodynamic trends are identified, the kinetic barriers and dynamic trajectories are not explored using ab initio molecular dynamics, potentially overlooking entropic contributions or short-lived intermediates. Third, the current analysis is restricted to eight specific gas-phase species; reactivity toward other radical species, photolytic processes, or competing anion-cation interactions remains to be investigated. Finally, the generalizability of the proposed framework linking electrostatic compatibility and bond lability to broader classes of carbon-centered radicals requires further experimental validation.

## Resource availability

### Lead contact

Further information and requests for resources and materials should be directed to and will be fulfilled by the lead contact, Junxi Liang (xbmujxliang@126.com).

### Materials availability

This study did not generate new unique reagents.

### Data and code availability

• Data reported in this paper will be shared by the lead contact upon request.
• This paper does not report original code.
• Any additional information required to reanalyze the data reported in this paper is available from the lead contact upon request.

## Acknowledgments

This work was financially supported by the Fundamental Research Funds for the Central Universities (31920260091, 31920250018, 31920240047, 2023SXZY-01), the 10.13039/501100001809National Natural Science Foundation of China (22468045 and 22165025), Gansu Provincial Department of Education: Industrial Support Plan Project (2024CYZC-04), Key Project of 10.13039/501100004775Natural Science Foundation of Gansu Province (25JRRA037), Chemistry Innovation Team of 10.13039/501100017619Northwest Minzu University in 2024 (1001660139 and 1001660141), Gansu Provincial Talent Program (2026QNTD011). We thank Dr. Lu Zhibin (Chinese Acad Sci, Lanzhou Inst Chem Phys, State Key Lab Solid Lubricat) for technical assistance.

## Author contributions

J.L.: resources, supervision, validation, and writing–review and editing. J.G.: investigation, software, and writing – original draft. X.W.: resources and software. Y.Z.: resources and software. X.B.: formal analysis. N.Y.: formal analysis. H.W.: resources, software. Y.S.: resources and software. P.J.: resources and software. Y.W.: resources and software. Q.S.: resources, software, and writing – review and editing. All authors read and approved the final manuscript.

## Declaration of interests

The authors declare no competing interests.

## STAR★Methods

### Key resources table

**Table:** 

REAGENT or RESOURCE	SOURCE	IDENTIFIER
**Software and algorithms**		

Materials Studio (Forcite, Amorphous Cell), Version 8.0	BIOVIA (Dassault Systèmes)^41^	https://so3.xwtingbin.com/s/materials-studio-computational-chemistry-software/
Gaussian 03, Revision A.1	Frisch et al.^42^	https://so3.xwtingbin.com/s/gaussian-quantum-chemistry-software/
ReaxFF CHONCl-2019 Force Field	Wolf et al.,^43^ Weismiller et al.,^44^ Senftle et al.^45^	N/A

### Method details

#### Model construction

Initial structural models were constructed using the Amorphous Cell module in Materials Studio (MS).^41^ A spherical CHCl˙^–^ anion cluster with a diameter of 60 Å was first generated. A 1 Å-thick adsorption layer composed of target gas molecules (O_2_, CO, CO_2_, NO, N_2_O, NO_2_, COS, or SO_2_) was subsequently deposited onto the cluster surface to simulate the gas-anion interface.^46^ To evaluate the dependence of gas-anion interactions on CHCl˙^–^ concentration, a series of 18 cubic simulation cells were constructed, each containing 100 molecules of a single gas species interacting with varying numbers of CHCl˙^–^ anions (10, 20, 30, up to 800).

#### MD simulations

All classical MD simulations were performed using the Forcite module with the COMPASS III force field.^47^^,^^48^^,^^49^^,^^50^ Electrostatic interactions were computed via the group summation method, while van der Waals (vdW) interactions were evaluated using the atom-based method.^51^^,^^52^ It is noted that the COMPASS III force field does not account for open-shell effects, e.g., polarization or charge transfer, therefore, these results are interpreted qualitatively to guide DFT initialization and validate bimolecular reactivity trends.

The simulation protocol for obtaining the most stable structures proceeded as follows:

Geometry optimization: Initial configurations underwent energy minimization.

Annealing: Systems were subjected to simulated annealing comprising 10 cycles, each consisting of 10^5^ steps.^53^

Density determination: Starting from the minimum-energy configuration post-annealing, an NPT ensemble MD simulation was conducted at 298.15 K and 1 atm for 5 ns with a 1 fs time step. The average density from the last 2 ns was adopted for subsequent simulations.

Equilibration: Systems were equilibrated under the NVT ensemble for 5 ns.^54^

Production run: A final 1 ns production run was executed under the same NVT conditions.

Final equilibrium configurations are provided in Figure S1.

#### Reactive force field (ReaxFF) validation

To model bond-breaking and formation, ReaxFF simulations were performed using the CHONCl-2019 force field, trained for halogen-carbon and halogen-oxygen chemistry.^43^^,^^44^^,^^45^ The force field’s reliability was validated by comparing ReaxFF-predicted reaction energies (e.g., CHCl˙^–^ + O_2_ → products) against our BHandHLYP/aug-cc-pVDZ quantum chemical calculations, yielding deviations within 5–8 kJ/mol.

#### DFT calculations

To elucidate reaction mechanisms and selectivity, potential energy surface (PES) calculations were performed using Gaussian 03.^42^ Geometries were fully optimized at the BHandHLYP/aug-cc-pVDZ level of theory.^55^^,^^56^^,^^57^^,^^58^^,^^59^^,^^60^^,^^61^ This functional/basis set combination was selected based on its proven accuracy for radical-anion reactions and gas-phase cluster anions compared to CCSD(T) benchmarks.^62^^,^^63^^,^^64^ No empirical dispersion correction was applied, as interactions are dominated by electrostatics rather than long-range vdW forces.^65^^,^^66^ The nature of stationary points was confirmed via harmonic vibrational frequency analysis. Intrinsic Reaction Coordinate (IRC) calculations were performed to trace pathways between transition states and connected minima.^67^ To refine energetics, higher-level single-point energy calculations were conducted at the QCISD(T)/aug-cc-pVDZ level using the BHandHLYP-optimized geometries. Equilibrium geometries are provided in Figure S2 and Table S1. Relative energies reported in PES profiles correspond to QCISD(T)/aug-cc-pVDZ//BHandHLYP/aug-cc-pVDZ results (Table S2). Structures are labeled as X-R (reactant), X-In (intermediate), X-Tn (transition state), and X-Pn (product), where X denotes the specific gas species and n is an Arabic numeral indicating distinct structures.

### Quantification and statistical analysis

#### Radial distribution functions (RDF)

Calculated using Equation 1,^68^ where *g*(*r*) is the RDF, *ρ* is the bulk number density, and *n*(*r*) is the number of particles within a spherical shell of radius *r* and thickness Δ*r.*

(Equation 1) $$g\left(r\right)=\left\langle \frac{n\left(r,r+dr\right)}{4\pi r^{2}\rho dr}\right\rangle$$

Local coordination numbers (*N*_*coord*_) were derived by integrating the first peak of the RDFs (Equation 2).

(Equation 2) $$N_{coord}=4\pi \rho \int_{r_{1}}^{r_{2}}r^{2}g\left(r\right)dr$$

#### Intermediate scattering function F(q,t)

Used to distinguish dynamical regimes; a decay to a non-zero plateau (F(q,∞)) indicates spatial localization/confinement.^69^

#### Mean squared displacement (MSD)

Calculated via Equation 3 to derive diffusion coefficients (*D*).^70^

(Equation 3) $$D=\frac{1}{6}\lim_{t-\infty }\frac{d}{dt}MSD\left(t\right)$$

## Supplemental information

Document S1. Figures S1 and S2

Table S1. Molecular coordinates obtained from DFT-BHandHLYP/aug-cc-pVDZ level

Table S2. Electronic structure energies for the reactions of CHCl˙^–^ + X (X = O2, CO, CO2, NO, NO2, N2O, SO2, and COS) stationary points (Total energy ET (a.u.), Zero-point energy EZPE (a.u.), and Relative energy ER (kJ mol-1))

## Footnotes

Supplemental information can be found online at https://doi.org/10.1016/j.isci.2026.117520.
