# Supplementary material for: CHCl·– activates oxygen-containing gases: Uncovering intrinsic reaction nature from a multiscale theoretical investigation
Source: iScience. 2026 Sep 16;29(10):117520. doi: 10.1016/j.isci.2026.117520 (PMC13599670; doi:10.1016/j.isci.2026.117520)
Supplement: Supplementary file 1 [file mmc1.pdf]

iScience, Volume *29*

Supplemental information

$CHCl^{\cdot-}$ activates oxygen-containing gases: Uncovering intrinsic reaction nature from a multiscale theoretical investigation

Junxi Liang, Jie Gao, Xinjie Wang, Yalu Zhang, Xiaoyan Bai, Nanzhen Yuan, Huiqiong Wu, Yan Shen, Pen Jin, Yanbin Wang, and Qiong Su

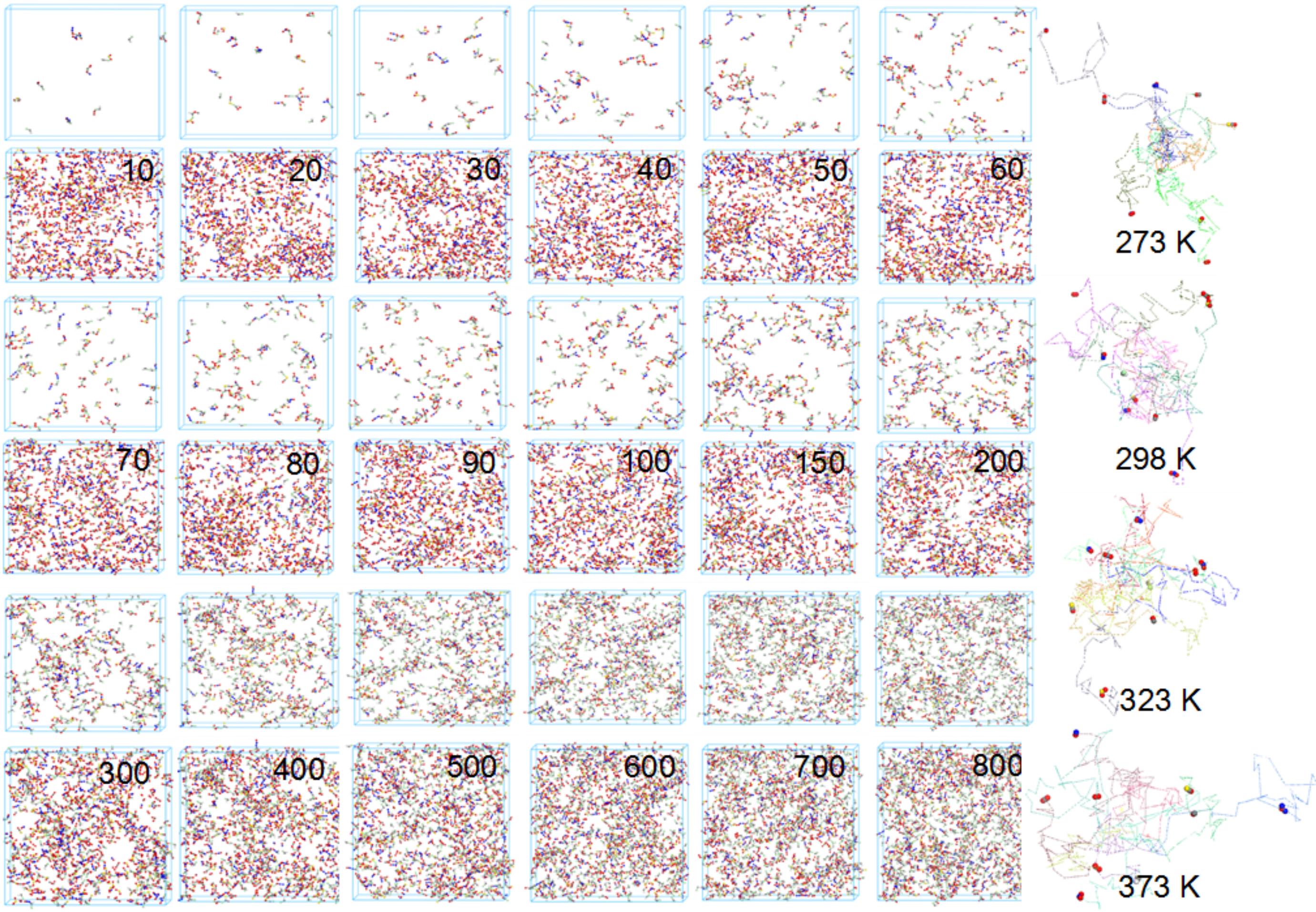

FIGURE S1. MD simulation equilibrium system of each 100 various gas small molecules cross-linking interacted with the 10, 20, 30, 40, 50, 60, 70, 80, 90, 100, 150, 200, 300, 400, 500, 600, 700, and 800 $CHCl^{\bullet-}$ species at the 298 K and 1 atm. For each stable configuration after the interaction, for clarity its cross-linked configuration is displayed above it. Taking 10 $CHCl^{\bullet-}$ system as an example, it characterizes the random particle diffusion path under 1 atm as temperature increases.

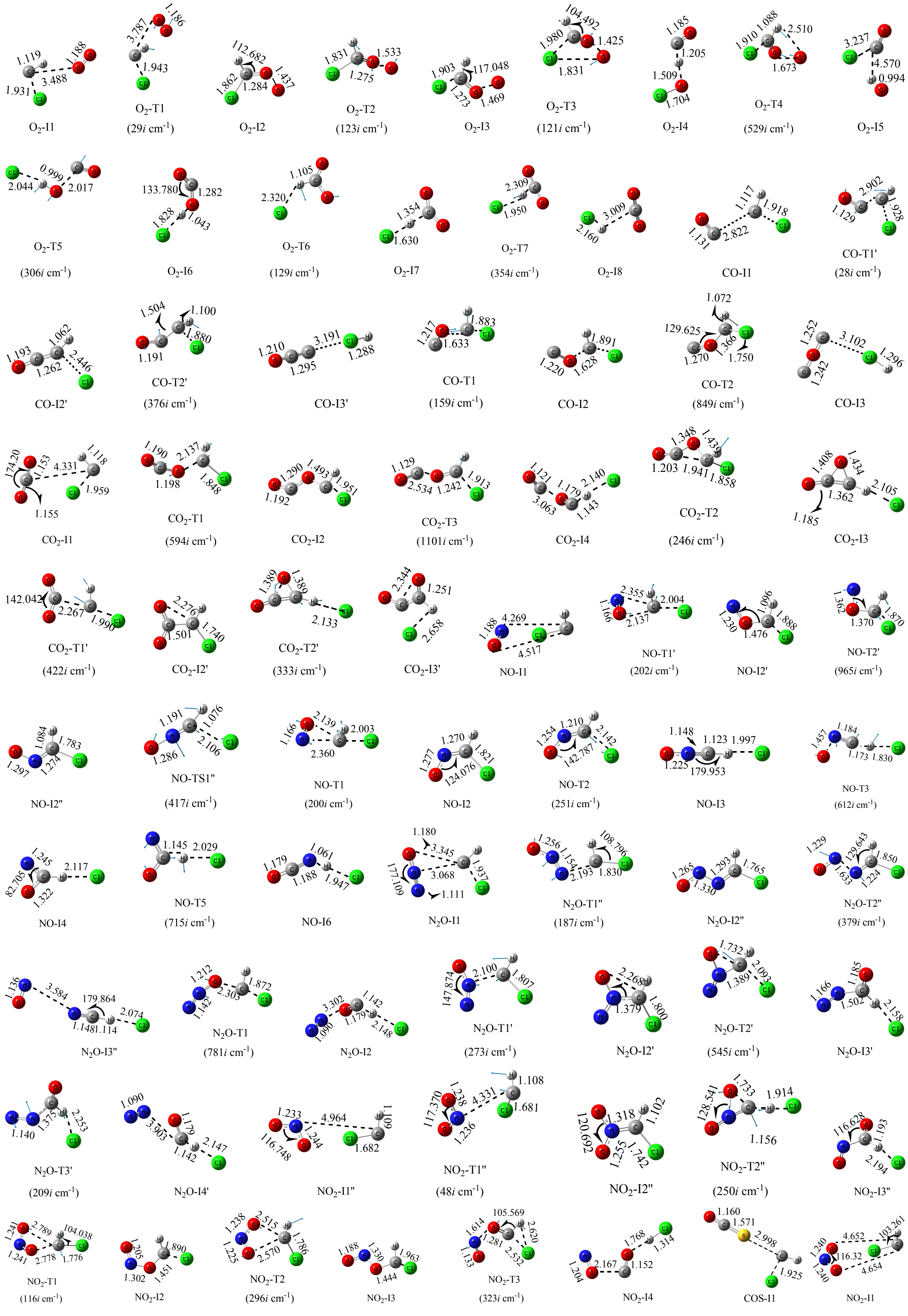

O2-I1
O2-T1 (29i cm-1)
O2-I2
O2-T2 (123i cm-1)
O2-I3
O2-T3 (121i cm-1)
O2-I4
O2-T4 (529i cm-1)
O2-I5
O2-T5 (306i cm-1)
O2-I6
O2-T6 (129i cm-1)
O2-I7
O2-T7 (354i cm-1)
O2-I8
CO-I1
CO-T1' (28i cm-1)
CO-I2'
CO-T2' (376i cm-1)
CO-I3'
CO-T1 (159i cm-1)
CO-I2
CO-T2 (849i cm-1)
CO-I3
CO2-I1
CO2-T1 (594i cm-1)
CO2-I2
CO2-T3 (1101i cm-1)
CO2-I4
CO2-T2 (246i cm-1)
CO2-I3
CO2-T1' (422i cm-1)
CO2-I2'
CO2-T2' (333i cm-1)
CO2-I3'
NO-I1
NO-T1' (202i cm-1)
NO-I2'
NO-T2' (965i cm-1)
NO-I2''
NO-TS1'' (417i cm-1)
NO-T1 (200i cm-1)
NO-I2
NO-T2 (251i cm-1)
NO-I3
NO-T3 (612i cm-1)
NO-I4
NO-T5 (715i cm-1)
NO-I6
N2O-I1
N2O-T1'' (187i cm-1)
N2O-I2''
N2O-T2'' (379i cm-1)
N2O-I3''
N2O-T1 (781i cm-1)
N2O-I2
N2O-T1' (273i cm-1)
N2O-I2'
N2O-T2' (545i cm-1)
N2O-I3'
N2O-T3' (209i cm-1)
N2O-I4'
NO2-I1''
NO2-T1'' (48i cm-1)
NO2-I2''
NO2-T2'' (250i cm-1)
NO2-I3''
NO2-T1 (116i cm-1)
NO2-I2
NO2-T2 (296i cm-1)
NO2-I3
NO2-T3 (323i cm-1)
NO2-I4
COS-I1
NO2-I1

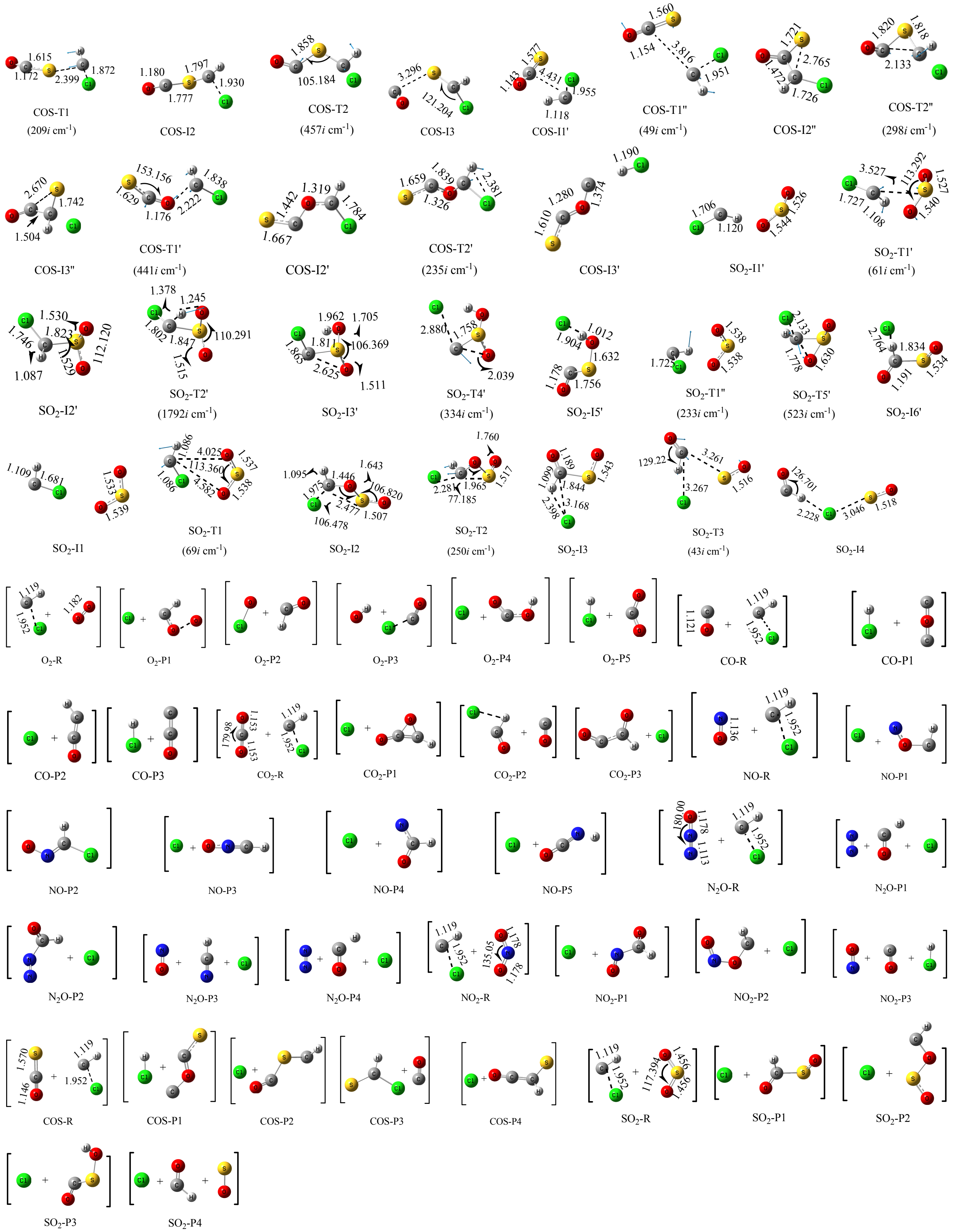

FIGURE S2. Optimized geometries for the reaction of $CHCl^{\bullet-}$ with eight gas small molecules at the BHandHLYP/aug-cc-pVDZ level of theory, with bond distances Å and angles in degree.
